# Data characterizing tensile behavior of cenosphere/HDPE syntactic foam

**DOI:** 10.1016/j.dib.2016.01.058

**Published:** 2016-02-04

**Authors:** B.R. Bharath Kumar, Mrityunjay Doddamani, Steven E. Zeltmann, Nikhil Gupta, Seeram Ramakrishna

**Affiliations:** a Lightweight Materials Laboratory, Department of Mechanical Engineering, National Institute of Technology Karnataka, Surathkal, India; b Composite Materials and Mechanics Laboratory, Mechanical and Aerospace Engineering Department, Tandon School of Engineering, New York University, Brooklyn, NY 11201, United States; c Center for Nanofibers & Nanotechnology, National University of Singapore, Singapore

**Keywords:** Tensile, Fly ash cenospheres, HDPE, Syntactic foam, Injectionmolding

## Abstract

The data set presented is related to the tensile behavior of cenosphere reinforced high density polyethylene syntactic foam composites “Processing of cenosphere/HDPE syntactic foams using an industrial scale polymer injection molding machine” (Bharath et al., 2016) [1]. The focus of the work is on determining the feasibility of using an industrial scale polymer injection molding (PIM) machine for fabricating syntactic foams. The fabricated syntactic foams are investigated for microstructure and tensile properties. The data presented in this article is related to optimization of the PIM process for syntactic foam manufacture, equations and procedures to develop theoretical estimates for properties of cenospheres, and microstructure of syntactic foams before and after failure. Included dataset contains values obtained from the theoretical model.

## Specifications table

**Table:** 

Subject area	Mechanical Engineering, Material Science
More specific subject area	Material Science/Mechanics of Composite Materials
Type of data	Tables, raw data in MS Excel files, optical camera images and scanning electron micrographs
How data was acquired	Data was acquired by experimental techniques
Data format	Raw and analyzed
Experimental factors	Optimization of pressure and temperature in polymer injection molding machine and composition of the composite material
Experimental features	Tensile properties and microstructure of injection molded syntactic foams. Modulus, ultimate tensile strength (UTS), elongation at UTS, fracture strain and fracture strength are the key properties determined
Data source location	Surathkal, India; Brooklyn, NY, USA
Data accessibility	Data is available in MS Excel format with this article

## Value of the data

• The tensile test results on syntactic foams produced by industrial scale injection molding machine are provided. The syntactic foams utilize fly ash cenospheres as filler material.
• The experimental results on tensile testing can be used by industry professionals for development of syntactic foams for specific applications.
• Theoretical models presented in the work can help researchers and industry professionals in predicting the properties of various compositions of syntactic foams and reduce experimentation.
• The data can be used in design and evaluation of consumer products for manufacture with this lower-cost lightweight material.
• Optimization data on industrial scale machine for syntactic foam manufacture can help other industries to adopt similar practices.

## 1 Data

Data presented in the article is pertaining to injection molding of fly ash cenosphere reinforced thermoplastic syntactic foam. High density polyethylene (HDPE) is used as the matrix material [1]. Cenospheres, which are an environmental pollutant, replace expensive HDPE in developing the syntactic foam components. The data contained in this brief consists of properties of raw materials, injection machine parameters, tensile test raw data and processed results, and micrographs of the material. In addition, images of prototype components are also provided.

## 2 Experimental design, materials and methods

### 2.1 Test equipment

Tensile testing was performed using a Zwick/Roell Z020 UTM with a 20 kN load cell in displacement control mode. Constant crosshead displacement rate is maintained at 50 mm/min. Stress and strain are calculated from load and displacement data.

Scanning electron microscope (JSM 6380LA, JEOL, Japan) is used for microstructural analysis. All the samples are sputter coated using JFC-1600 auto fine coater (JEOL, Japan).

Nikon D7000 camera with Nikkor 35 mm *f*/1.8 and Tokina AT-X Pro 100 mm *f*/2.8 macro lens are used for imaging fractured features.

### 2.2 Raw materials

The syntactic foams tested in this work were fabricated using high density polyethylene (HDPE, Reliance Polymers, Mumbai, India) as the matrix and fly ash cenospheres (Censosphere India Pvt. Ltd., Kolkata, India) as the hollow filler. Chemical and sieve analyses of the cenospheres are shown in Table 1.

### 2.3 Syntactic foam fabrication method

The constituents are mechanically mixed before being fed into a polymer injection molding machine and molded into ASTM D638-conforming tensile bars. The specifications of the injection molding machine are provided in Table 2 and the process parameters are presented in Table 3. Syntactic foams containing 0% (HDPE), 20% (HDPE20), 40% (HDPE40), and 60% (HDPE60) by weight of cenospheres are fabricated.

### 2.4 Micrography

**Fig. 1:**
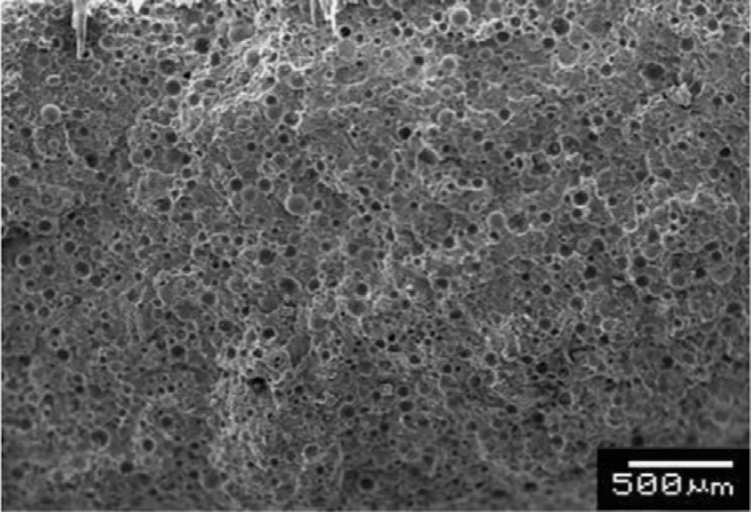
A scanning electron micrograph of a representative HDPE60 specimen on freeze-fractured surface.

Fig. 1 shows a micrograph of HDPE60. Uniform dispersion of the hollow spheres in the HDPE matrix is observed in this micrograph, which affirms the feasibility of manufacturing syntactic foams using injection molding at high filler loadings (up to 66.4 vol% of cenospheres).

### 2.5 Tensile characterization

**Fig. 2:**
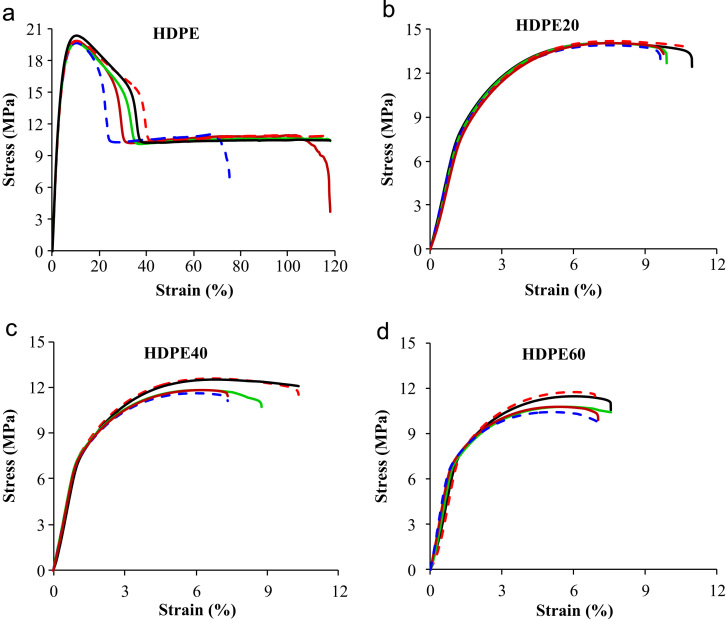
Stress–strain graphs of (a) neat HDPE and syntactic foam specimens with (b) 20%, (c) 40% and (d) 60% by weight of cenospheres.

The load and displacement data are acquired from the tensile test. These data are used to calculate the stress–strain curves for each specimen shown in Fig. 2. The data used to produce these curves are included in the data folder (Tensile-Data.xlsx). This data can be used to calculate the tensile modulus, ultimate tensile strength (UTS), elongation at UTS, fracture strain and fracture strength. The modulus is calculated as the slope in the elastic region.

**Fig. 3:**
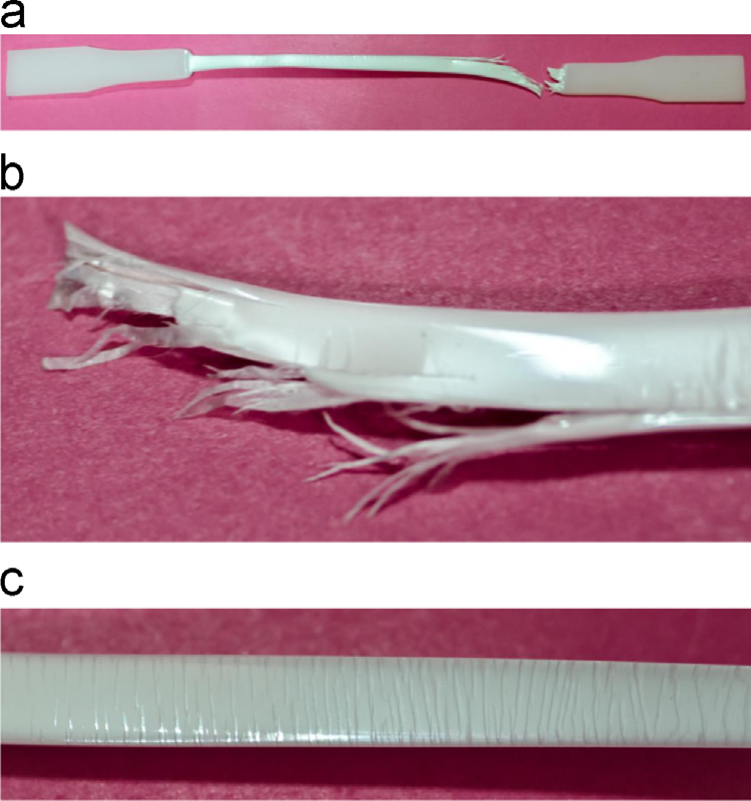
(a) A representative failed specimen neat HDPE under tensile loading, (b) the magnified failure region showing broom-like fracture and (c) deformation marks along the entire gauge length.

**Fig. 4:**
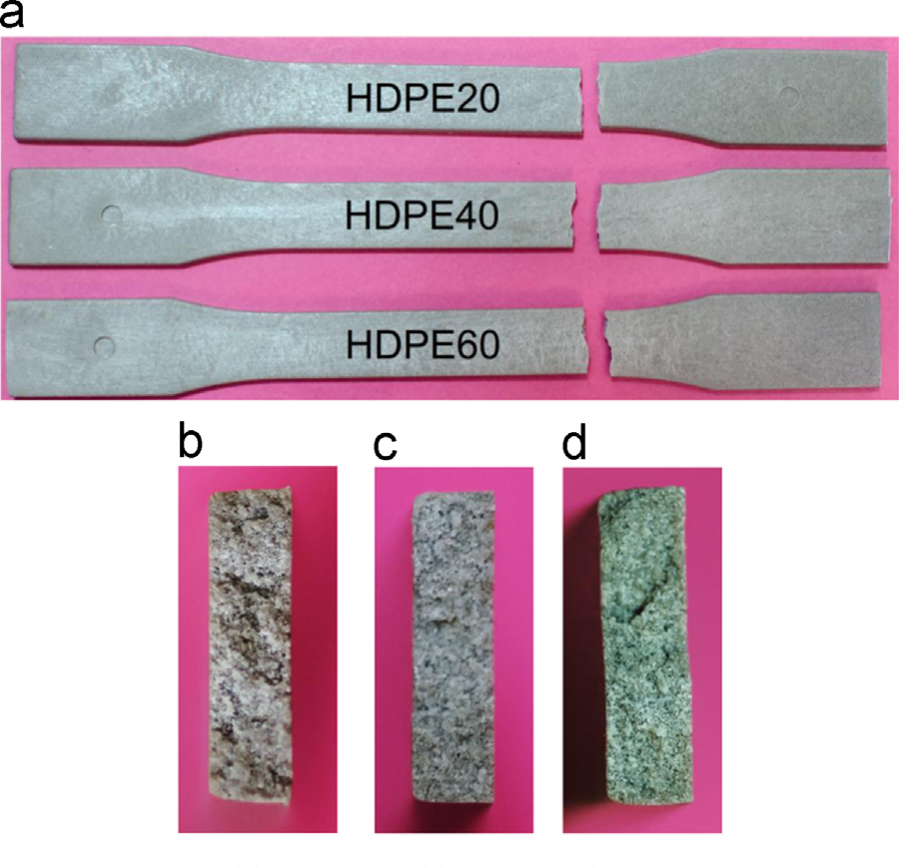
(a) Representative failed specimens of syntactic foams. Fracture surface of (b) HDPE20, (c) HDPE40 and (d) HDPE60 specimens. The fracture appears different from the fibrous fracture observed for the neat HDPE resin.

Failure features of neat HDPE specimen tested under tensile loading are presented in Fig. 3. This figure shows a broom-like fracture front and deformation marks along the entire specimen gauge length. Macroscopic failure features of syntactic foams are presented in Fig. 4, which can be compared with the failure appearance of the neat HDPE resin. The failure of syntactic foams appears to be relatively brittle with only a little plastic deformation, which can be confirmed from the tensile stress–strain data.

**Fig. 5:**
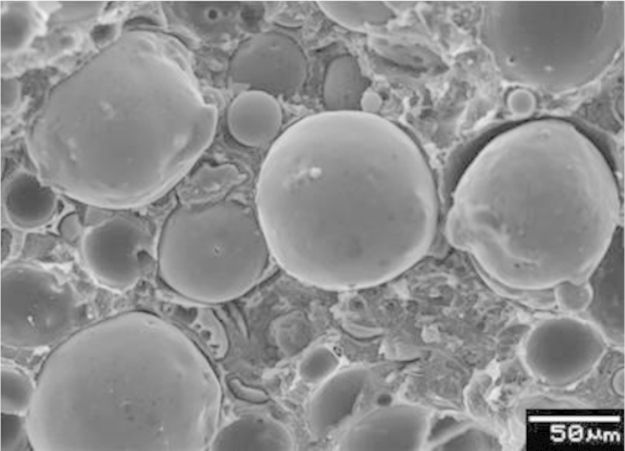
Fracture surface of HDPE40 syntactic foam at higher magnification showing intact particles and deformed matrix. The particle-matrix interfacial failure is also observed.

A large number of surviving particles after the tensile test are visible in Fig. 5 for an HDPE40 specimen. The crack propagation occurs mainly in the matrix resin. Defects in the cenosphere wall, non-spherical shape, and poor particle-matrix interfacial adhesion are observed in Fig. 5.

**Fig. 6:**
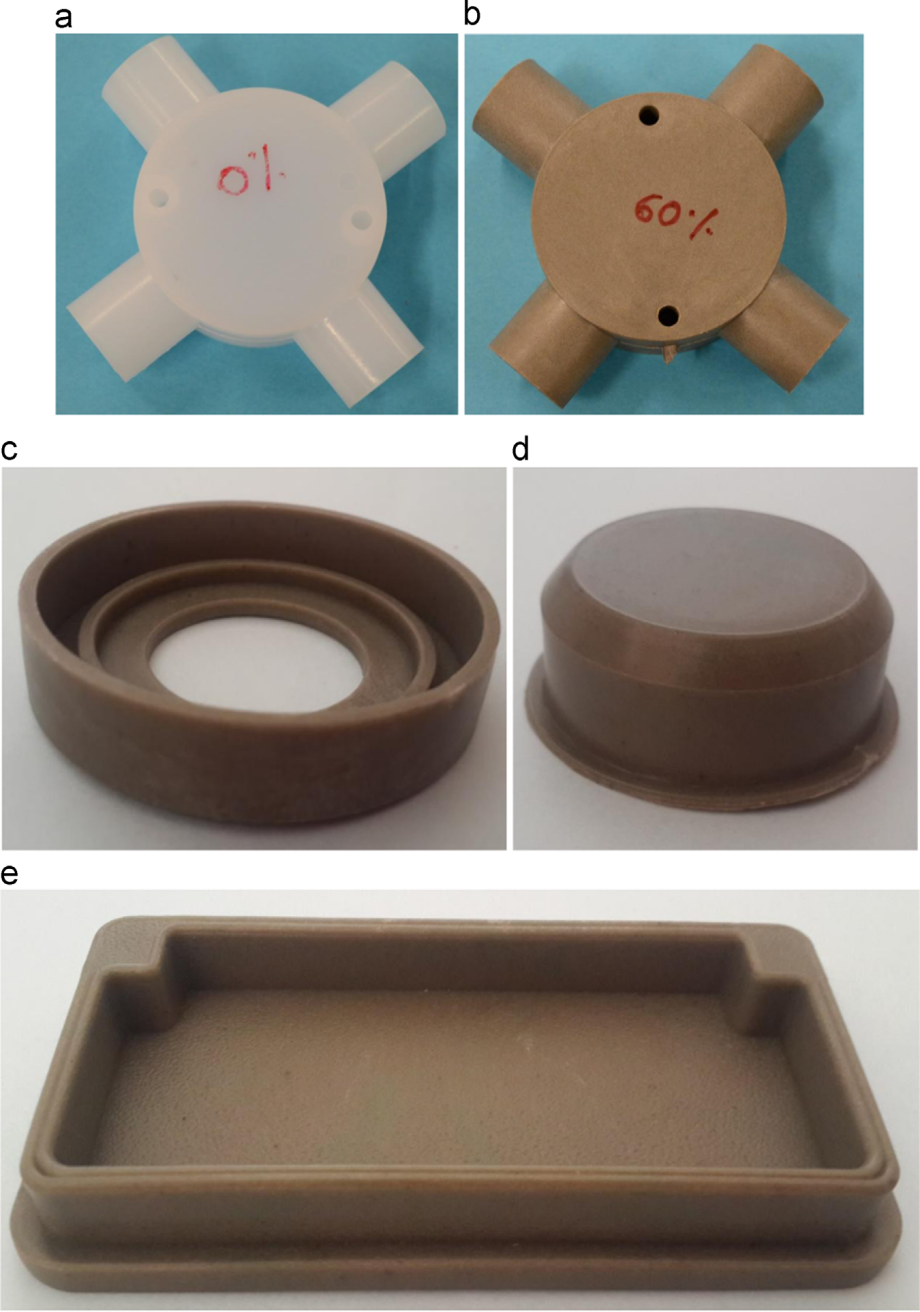
Prototype components cast in the study: (a) an example of an electrical junction box cast of pure HDPE, (b) the electrical junction box cast on the same machine with syntactic foam. Other syntactic foam prototypes: (c) a part of a ball bearing, (d) a bottle cap and (e) bottom cap of a chair leg.

The optimized parameters for the industrial scale polymer injection molding machine are used to cast prototype parts shown in Fig. 6. These parts include narrow sections, screw threads, and holes showing the possibility of casting complicated parts with syntactic foams.

### 2.6 Theoretical modeling

A theoretical model for syntactic foams is presented that is used in a parametric study to determine the effective properties of the cenospheres from the experimental data on syntactic foams. The Porfiri–Gupta model involves solving for an infinitely dilute dispersion and using a differential scheme to extend the results to high particle loading [2], [3]. The general expression of the differential scheme for elastic modulus is given as

(1) $$\frac{dE}{E}=f_{E}(E_{c},\upsilon_{c},E_{m},\upsilon_{m},\eta )\frac{d\Phi_{f}}{1-\Phi_{f}/\Phi_{m}}$$

where *E*_*c*_ and *ν*_*c*_ are Young׳s modulus and Poisson׳s ratio of the ceramic particle wall, and *E*_*m*_ and *ν*_*m*_ are the modulus and Poisson׳s ratio of the matrix material. In addition, *Φ*_*f*_ represents cenospheres volume fraction and *Φ*_*m*_ denotes the maximum packing factor of particles, taken to be 0.637, which represents the random packing factor of equal size spheres. The parameter *η* is the radius ratio of the hollow particles, defined as the ratio of the inner radius to outer radius. The full formulation of the model is available in [2]. A parametric study is conducted by numerically solving the model for different input parameters and comparing with the experimental results to estimate the properties of cenospheres. A set of solutions for the elastic modulus as a function of the volume fraction of particles are given in the included data (Porfiri–Gupta-Model.xlsx).

As the properties of the ceramic wall material needed for the model are not known, they are estimated using the approach demonstrated by [4] using data for the particular grade of particles used in fabrication of the composites in this work (Table 1). The presence of minor constituents is ignored, and the properties of the major constituents are taken as shown in Table 4. The elastic modulus of the ceramic wall material is found by

(2) $$E_{c}=\sum_{i}\phi_{i}E_{i}$$

where *ϕ*_*i*_ and *E*_*i*_ are the volume fraction and elastic modulus of each component. Density and Poisson׳s ratio can also be found using equations of similar type. The ceramic properties obtained by this method are presented in Table 4. Assuming that the cenosphere wall thickness is uniform and is fully dense, the value of *η* can be determined by

(3) $$\eta =\sqrt[{3}]{1-\frac{\rho_{TPD}}{\rho_{c}}}$$

where $\rho_{TPD}$ is the true particle density and $\rho_{c}$ is the density of the ceramic. The value of *η* is found to be 0.90 for the cenospheres used in these composites.

The approaches used in determining the properties of the cenospheres ignore the presence of defects in the walls and thus lead to higher predictions than the actual modulus. In order to obtain an estimate of the effective properties of the cenospheres with defects, a parametric study is conducted using the theoretical model. In the first step the cenosphere wall modulus obtained from the rule of mixtures is used while the radius ratio is varied to minimize the difference between the model and experimental data. Conversely the radius ratio obtained from density measurements is kept constant while the ceramic modulus parameter is varied to obtain good fitting with the experiments. This generates two sets of effective properties for the cenospheres: *E*_*c*_=157 MPa with *η*=0.995, and *E*_*c*_=7.5 GPa with *η*=0.9. The effective modulus of an equivalent solid sphere $\bar{E}$ is found using [5]

(4) $$\bar{E}=\frac{E_{c}(1-2\nu )(1-\eta^{3})}{(1-2\nu )+\left(\frac{1+\nu }{2}\right)\eta^{3}}$$

Both of the pairs of cenosphere properties found in the parametric study yield an effective modulus of 1.20 GPa due to the presence of defects in their walls and other irregularities. Eqs. (1), (2), (3) can be used to predict the properties of hollow particles and syntactic foams in a similar manner with other types of raw material.

## Figures and Tables

**Table 1:** Chemical, physical and sieve analysis details of cenospheres⁎.

**Physical properties**		**Chemical analysis (%)**		**Sieve analysis**	
True particle density	800 kg/m^3^	SiO_2_	52–62	+30 # (500 µm)	Nil
Bulk density	400–450 kg/m^3^	Al_2_0_3_	32–36	+ 60 # (250 µm)	Nil
Hardness (MOH)	5–6	CaO	0.1–0.5	+100 # (150 µm)	Nil
Compressive strength	180–280 kg/m^3^	Fe_2_0_3_	1–3	+120 # (125 µm)	Nil
Shape	Spherical	TiO_2_	0.8–1.3	+150 # (106 µm)	0–10%
Packing factor	60–65%	MgO	1–2.5	+240 # (63 µm)	70–95%
Wall thickness	5–10% of shell dia	Na_2_O	0.2–0.6	−240 #	0–30%
Color	Light gray–light buff	K_2_O	1.2–3.2		
Melting point	1200–1300 °C	CO_2_	70		
pH in water	6–7	N_2_	30		
Moisture	0.5% max				
Loss on ignition	2% max				
Sinkers	5% max				
Oil absorption	16–18 g/100 g				

⁎ As specified by supplier.

**Table 2:** Injection molding machine specifications.

**Machine**	**Parameters**	**Typical value**
General specifications	Make	Windsor, India
Capacity	80 ton
Injection Unit	Plasticizing capacity	40 kg/h
Capacity molded per shot barrel/screw unit with pressure on material	1020 kg/cm^2^, 110 cm^3^
Screw diameter	42 mm
Injection stroke	80 mm
Screw speed infinitely variable	0–200 rpm
Capacity of hopper	30 kg
Locking unit	Mold clamping force	80 ton
	Size of mold plates	500×500 mm
	Distance between tie bars	330×330 mm
	Maximum mold opening	450 mm
	Maximum mold thickness	150 mm

**Table 3:** Processing conditions for injection molded syntactic foam composites⁎.

**Parameters**	**Typical value**
Mold temperature (°C)	50–60
Nozzle temperature (°C)	160
Heating zone temperature (°C)	160
Screw speed (RPM)	30
Injection speed (mm/s)	18
Injection time (s)	4
Holding time (s)	6
Cooling time (s)	20
Total cycle time (s)	30

⁎ As specified by Konkan Speciality Polyproducts Pvt. Ltd., Mangalore, Karnataka, India.

**Table 4:** Material properties used in theoretical modeling.

**Material**	**Modulus (GPa)**	**Poisson׳s ratio**	**Density (kg/m**^**3**^**)**	**Mass fraction (%)**
SiO_2_	70	0.17	2650	62
Al_2_O_3_	370	0.22	3950	38
Cenosphere wall	157	0.19	3027	–
HDPE	0.529⁎	0.425	1056⁎	–

⁎ From experiments conducted in this study.
